# Archimedes navigation system-guided bronchoscopic trans-parenchymal nodule access for peripheral lung nodules: a single-center experience

**DOI:** 10.3389/fonc.2025.1628410

**Published:** 2025-09-10

**Authors:** Ping-Tsung Yu, Sheng Hsiung Yang, Hui-Hsuan Shih, Wei Ming Huang, Jie-Yang Jhuang, Mei-Lin Chan, Wen-Chien Huang

**Affiliations:** ^1^ Chest Division, Department of Internal Medicine, MacKay Memorial Hospital, Taipei City, Taiwan; ^2^ Department of Radiology, MacKay Memorial Hospital, Taipei City, Taiwan; ^3^ Department of Medicine, MacKay Medical College, New Taipei City, Taiwan; ^4^ MacKay Junior College of Medicine, Nursing, and Management, New Taipei City, Taiwan; ^5^ Department of Pathology, MacKay Memorial Hospital, New Taipei City, Taiwan; ^6^ Department of Thoracic Surgery, MacKay Memorial Hospital, Taipei City, Taiwan

**Keywords:** archimedes navigation system, bronchoscopic trans-parenchymal nodule access (BTPNA), diagnostic yield, peripheral pulmonary lesion (PPL), virtual bronchoscopic navigation (VBN)

## Abstract

**Background:**

Peripheral pulmonary lesions (PPLs) present diagnostic challenges because of their small size and distal airway location, often requiring invasive surgical biopsies. The Archimedes™ navigation system assists the bronchoscopic trans-parenchymal nodule access (BTPNA) procedure, offering a minimally invasive alternative for diagnosing PPLs.

**Methods:**

This retrospective study evaluated the safety and efficacy of BTPNA guided by the Archimedes™ navigation system in 10 patients with single PPLs (<20 mm) and no bronchus signs. Preoperative CT scans were used to create a 3D virtual airway model, identify an optimal point of entry (POE), and plan an avascular pathway to the nodule. The procedure combined virtual bronchoscopy, real-time fluoroscopy, and radial endobronchial ultrasound (R-EBUS). Tissue samples were collected for histopathological analysis, and complications were monitored post-procedure.

**Results:**

A total of 10 patients (median age: 54.5 years) were included, with a diagnostic yield of 70.0%. Successful diagnoses included adenocarcinoma *in situ*, granulomatous inflammation, and invasive adenocarcinoma. No complications were observed. The mean size of the nodular lesions in the 10 cases was 13.9 mm. Median distances were 12.9 mm from pleura to target lesion, and median tunnel length was 57.8 mm.

**Conclusions:**

The Archimedes™ system-guided BTPNA is safe and effective for diagnosing small PPLs without bronchus signs, demonstrating precise localization and tissue sampling while minimizing invasiveness. No complications were observed, highlighting the procedure’s safety. Larger prospective studies are warranted to validate these findings and refine patient selection and procedural techniques to optimize outcomes.

## Introduction

Peripheral pulmonary lesions (PPLs), typically small and located in distal airways, pose challenges for early lung cancer diagnosis due to their peripheral location and limited accessibility with conventional bronchoscopy (1). Although advances in imaging and biopsy procedures such as radial probe endobronchial ultrasound have improved detection rates, achieving a diagnostic sensitivity ranging between 69% to 73%, a substantial proportion of PPLs remain undiagnosed.

Virtual bronchoscopic navigation (VBN) has emerged as a valuable tool to overcome the challenges of accessing peripheral pulmonary lesions by using pre-acquired computed tomography (CT) imaging to create virtual pathways that guide the bronchoscope to hard-to-reach areas, thereby improving diagnostic accuracy—with an overall yield of 73.8%, including 67.4% for lesions ≤2 cm—and reducing the need for more invasive procedures (2–4). Diagnostic accuracy improves to 80.4 – 83.6% when VBN is combined with endobronchial ultrasound (EBUS), which enhances visualization of the lesion and surrounding structures, enabling more precise localization (5, 6). However, diagnosing some PPLs remains challenging in the absence of bronchus sign.

The bronchoscopic trans-parenchymal nodule access (BTPNA) is an advanced procedure facilitated by Archimedes™ navigation system, which have potential to fix these problems. Unlike conventional approaches that navigate through the pre-existed airways, BTPNA directly accesses lung parenchyma through newly created pathway, allowing accurate biopsy of PPLs (7–9). It processes pre-acquired CT scans to identify the optimal point of entry (POE) in the airway and delineates a safe, avascular path to the target lesion. During the procedure, this virtual pathway assists in guiding the bronchoscope, while real-time fluoroscopy overlays live imaging with the pre-planned route to ensure precise navigation (10, 11). By integrating these advanced techniques, the Archimedes system provides a minimally invasive method, that may be an alternative way to CT-guide biopsy or surgical methods for diagnosing PPLs without bronchus sign.

This study examines the procedural and diagnostic details of our experience with 10 cases of PPLs at a tertiary medical center, aiming to present the safety and diagnostic yield of BTPNA guided by the Archimedes navigation system in patients with PPLs without bronchus sign. The findings will contribute to the growing body of evidence supporting the use of BTPNA for the early and accurate diagnosis of PPLs.

## Methods

### Study design and patient selection

This retrospective, observational study evaluated the safety and diagnostic yield of the Archimedes™ VBN system combined with BTPNA for diagnosing PPLs at Taipei MacKay Memorial Hospital, Taiwan. The definition of success diagnostic yield is to reflect the concordance between the histopathological or cytopathological diagnosis from BTPNA-obtained specimens and the final surgical pathology. Patients aged 18 years or older who underwent the procedure between 2019 and 2022 were included. Eligible patients had CT-detected peripheral nodules <20 mm without bronchus signs and were suitable candidates for general anesthesia. Exclusion criteria included intolerance to bronchoscopy, coagulopathy, severe pulmonary disease, active infection, nodules located in the apical segment, or refusal of the procedure. Only patients who underwent video-assisted thoracoscopic surgery (VATS) after BTPNA were enrolled.

### Ethics statement

The study was reviewed and approved by the Institutional Review Board (IRB) of MacKay Memorial Hospital (Approval Number: 24MMHIS052e).

### Preoperative planning

For each patient, preoperative chest CT scans were uploaded into the Archimedes™ system (Broncus Medical, San Jose, CA, USA) to create a 3D virtual model of the thoracic anatomy, including airways, vascular structures, and the target nodule. The system identified an optimal POE on the airway wall and calculated an avascular pathway to the nodule. Virtual Doppler imaging highlighted vascular structures, minimizing the risk of vascular injury. Tunnel length (distance from the POE to the lesion), bronchial diameter, and the nodule’s proximity to the pleura, were assessed and recorded. A virtual bronchoscopy simulation confirmed the navigation plan’s feasibility.

#### Procedure

##### BTPNA with archimedes navigation

Procedures were performed under general anesthesia with patients in the supine position. An endotracheal tube was used for ventilation. The bronchoscope was guided to the designated POE using the Archimedes™ system (Broncus Medical), assisted by real-time fused fluoroscopy and navigation.

An 18-gauge FleXNeedle^®^ 10005 - 2 (Broncus Medical) was used to puncture the airway wall at the POE, followed by balloon dilation of the tunnel. A blunt-tipped stylet and sheath were then advanced through the POE under fluoroscopic guidance along the pre-planned pathway to the nodule.

During advancement of the sheath, fluoroscopic images were reviewed every 10 – 20 mm to ensure proper alignment and avoid complications. After the sheath reached the proximal edge of the nodule, cone-beam CT was used to confirm its location. The stylet was then removed, and biopsy forceps were used to collect tissue samples, with an average of four samples taken per patient.

#### Detailed technique of BTPNA using the archimedes navigation system

BTPNA procedures were performed in a hybrid operating room equipped with real-time fluoroscopy and cone-beam computed tomography (CBCT), enabling precise intraoperative imaging guidance. Prior to the procedure, patient-specific 3D models and access pathways to the target lesions were generated using the Archimedes™ Virtual Bronchoscopic Navigation System (Broncus Medical, San Jose, CA, USA) based on high-resolution chest CT.

Navigation began with a thin bronchoscope (Olympus BF-P190, Olympus Corporation, Tokyo, Japan) to localize the airway near the planned entry point, followed by the use of a therapeutic bronchoscope (Olympus BF - 1TQ290) for instrument delivery, due to its larger working channel (2.8 mm). The transparenchymal access was established using the Archimedes-specific toolkit, including the Transparenchymal Access Tool (TPA), a needle and guidewire set, and a dedicated triple-lumen sheath system. The sheath was advanced under real-time fluoroscopic guidance along the predefined trajectory. Once the access sheath was positioned, intraoperative CBCT imaging was performed to confirm the sheath tip location relative to the pulmonary nodule.

Tissue sampling was then performed via the sheath’s working channel using standard biopsy forceps (e.g., Olympus FB - 231D or equivalent), allowing for multiple passes to obtain adequate specimens. The integration of real-time navigation, fluoroscopic guidance, and CBCT confirmation ensured accurate targeting, minimized parenchymal injury, and optimized diagnostic yield in peripheral nodules lacking bronchus sign.

Following forceps biopsy via the transparenchymal access sheath, all obtained specimens were immediately subjected to rapid on-site evaluation (ROSE) to assess sample adequacy. ROSE serves primarily as an on-site tool to assess specimen adequacy and provides preliminary cytological impressions. The tissue was gently smeared or imprinted onto glass slides and air-dried. Slides were stained using the Diff-Quik method, a modified Romanowsky stain consisting of a fixative (methanol), a solution containing eosinophilic dye (Solution I), and a basophilic dye (Solution II). This quick staining procedure (typically <2 minutes) allowed cytotechnologists or on-site cytopathologists to evaluate cellularity, specimen quality, and preliminary diagnostic features under a light microscope. ROSE provided real-time feedback during the procedure, allowing additional samples to be taken immediately if the initial specimen was deemed insufficient, thereby improving overall diagnostic yield and procedural efficiency.

### Histological evaluation and postoperative management

Cytology results from BTPNA were assessed using ROSE, followed by standard histopathological examination of specimens obtained via VATS, processed at the Department of Pathology. Diagnostic success was defined as the concordance between ROSE and postoperative histopathological examination results.

Post-procedural chest X-rays were done by fluoroscopy to assess for pneumothorax or pneumomediastinum. Patients were closely monitored for hemodynamic stability, oxygen saturation, and any signs of significant bleeding.

### Statistical analysis

In the comparison of clinical characteristics between patients with successful and unsuccessful diagnoses, categorical variables are presented as counts (percentage) and analyzed by Fisher’s exact test. Continuous variables are presented as median (IQR) or mean and analyzed using the Wilcoxon Rank Sum test. A two-sided p-value of <0.05 was established as statistical significance and all statistical analyses were performed using the statistical software SAS version 9.4 (SAS Institute Inc., Cary, NC, USA).

## Results

### Clinical characteristics of included patients

**Figure 1:**
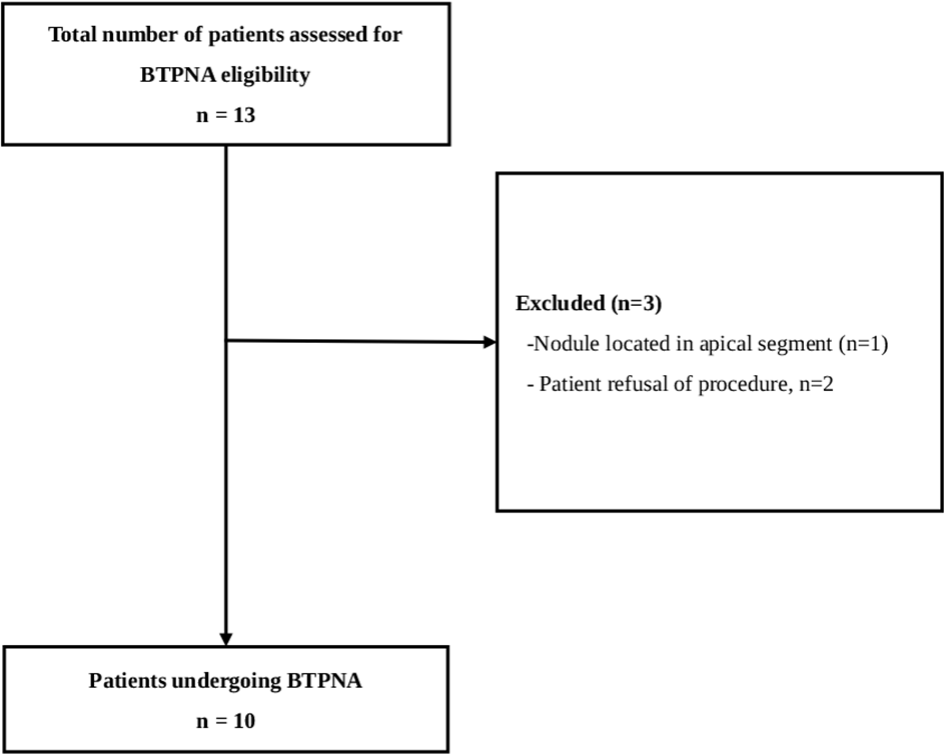
Patient enrollment flowchart.

Data of all 10 included patients with single PPLs (<20 mm) and no bronchus signs were included in the analysis (**Figure 1**). The median age was 54.5 years, and half of the patients (50.0%) were female. Fluoroscopy visibly identified the lesion in 1 patient (10.0%). Most patients (60.0%) had lesions measuring less than 15 mm in size with the right upper lobe being the most common lesion location (50.0%). The mean size of the nodular lesions was 13.9 mm.

The median distances from pleura to target lesion was 12.9 mm, and from carina to POE was 100.0 mm, respectively. Additionally, the median values for the final airway diameter at the midpoint, tunnel length, and distance to vessels were 6.9 mm, 57.8 mm, and 3.3 mm, respectively. The median lung volume was 3.9 L (**Table 1**).

**Table 1:** Clinical characteristics of patients with successful and unsuccessful diagnoses.

Clinical characteristics	All patients (n= 10)	Unsuccessful diagnoses (n= 3)	Successful diagnoses (n= 7)	*p-value*
Age, years	54.5 (50.0 - 70.0)	62.0 (50.0 - 70.0)	54.0 (47.0 - 73.0)	0.819
Sex				>0.999
Male	5 (50.0)	2 (66.7)	3 (42.9)	
Female	5 (50.0)	1 (33.3)	4 (57.1)	
Visible on fluoroscopy	1 (10.0)	1 (33.3)	0 (0.0)	0.300
Lesion size, mm				0.200
<13	4 (40.0)	0 (0.0)	4 (57.1)	
>=13	6 (60.0)	3 (100.0)	3 (42.9)	
Lesion location				0.167
RLL	5 (50.0)	1 (33.3)	4 (57.1)	
RUL	1 (10.0)	0 (0.0)	1 (14.3)	
LLL	2 (20.0)	0 (0.0)	2 (28.6)	
LUL	2 (20.0)	2 (66.7)	0 (0.0)	
Pleura to target, mm	12.9 (8.7 - 15.0)	12.6 (8.7 - 15.0)	13.1 (7.3 - 16.5)	>0.999
Carina to POE, mm	100.0 (86.0 - 114.0)	86.0 (70.0 - 99.0)	101.0 (89.0 - 119.0)	0.170
Final airway diameter, mm				
Minimum	5.2 (4.4 - 7.2)	6.0 (5.2 - 7.2)	4.8 (4.0 - 8.0)	0.423
Midpoint	6.9 (5.1 - 10.0)	10.0 (7.8 - 12.3)	6.3 (5.0 - 7.1)	0.067
Maximum	6.1 (5.6 - 8.6)	8.6 (6.5 - 9.2)	5.8 (4.7 - 6.5)	0.136
Tunnel length, mm	57.8 (55.0 - 61.5)	57.0 (24.5 - 60.0)	58.5 (55.0 - 66.5)	0.494
Distance to vessels, mm	3.3 (2.9 - 4.9)	3.6 (3.2 - 33.0)	3.2 (2.7 - 4.9)	0.304
Lung volume, L	3.9 (3.4 - 4.4)	3.9 (2.8 - 4.6)	3.9 (3.4 - 4.4)	>0.999

Continuous variables with normal distribution are presented as median (IQR); categorical variables are presented as counts (percentage).

RLL, right lower lobe; RUL, right upper lobe; LLL, left lower lobe; LUL, left upper lobe; POE, point of entry.

P-values < 0.05 are shown in bold.

### Comparison of characteristics between patients with successful and unsuccessful diagnoses

The overall rate of successful diagnosis was 70.0% (n=7). No statistically significant differences such as lesions size, pleura to target, carina to POE or final airway diameter were observed in other variables between the two groups (**Tables 1**, **2**).

**Table 2:** Patient data, results of ROSE, pathology reports and complications.

No.	Age, years	Sex	Lung segments	Site	Nodule type	Nodule size^*^, mm	Tunnel length, mm	Final airway diameter, mm	Carina to POE distance, mm	Pleura to target, mm
1	55	M	Segment 10 of the right lung	RLL	Part solid	11	61.5	5.2-6.3	114	7.3
2	47	F	Segment 10 of the center lung	LLL	Pure GGO	12	66.5	3.7-5.1	101	13.1
3	46	M	Segment 8 of the right lung	RLL	Pure GGO	11	48.5	4.0-7.1	89	2.4
4	70	M	Combined segments 1 and 2 of the center lung	LUL	Pure GGO	16	57.0	5.2-7.8	86	15.0
5	79	M	Segment 9 of the center lung	LLL	Solid	14	58.5	4.8-6.7	119	14.1
6	54	F	Segment 1 of the right lung	RUL	Part solid	14	56.5	8.5-11.1	39	8.7
7	54	F	Segment 9 of the right lung	RLL	Pure GGO	8	55.0	4.4-5	101	16.5
8	62	F	Segment 3 of the center lung	LUL	Pure GGO	18	60.0	6.0-12.3	70	8.7
9	50	M	Segment 9 of the right lung	RLL	Solid	20	24.5	7.2-10.0	99	12.6
10	73	F	Segment 8 of the right lung	RLL	Pure GGO	15	82.0	8.0-5.0	167	17.0

ROSE, Rapid On-Site Evaluation; M, male; F, female; Y, yes; N, no; AIS, adenocarcinoma *in situ*; AAH, atypical adenomatous hyperplasia; GGO, ground glass opacity; mm, millimeter; RLL, Right Lower Lobe; LLL, Center Lower Lobe; RUL, Right Upper Lobe; LUL, Center Upper Lobe; POE, point of entry.

^*^ Mean nodule lesion size was 13.9 mm.

### ROSE, pathology reports, and complications in all patients

Among the 10 patients, six (60.0%) had lesions located in the right lung and four (40.0%) in the left lung. ROSE reported benign findings in two patients (20.0%), inflammation in three patients (30.0%), atypia in two patients (20.0%), and adenocarcinoma cell in three patients (30%). Final pathology confirmed chronic inflammation in one patient (10.0%), anthracosis in one patient (10.0%), granulomatous inflammation in two patients (20.0%), AIS in four patients (40.0%), and invasive adenocarcinoma in two patients (20.0%). No complications were observed (**Tables 2**, **3**).

**Table 3:** Patient data, results of ROSE, pathology reports and complications (cont.).

No.	Visible on fluoroscopy	ROSE via BTPNA	Pathology of specimen via BTPNA	Pathology via VATS	Correlation with resection specimens	Complications
1	N	Inflammatory cells	Chronic inflammation	Chronic granulomatous inflammation	YES	None
2	N	Atypical cells	Atypia	Anthracosis	YES	None
3	N	Malignant cells	at least adenocarcinoma in situ	AIS	YES	None
4	N	Atypical cells	Atypia	AIS	No	None
5	N	Inflammatory cells	Inflammation	Chronic inflammation	YES	None
6	N	Malignant cells	Adenocarcinoma	Invasive adenocarcinoma	YES	None
7	N	Inflammatory cells	Granulomatous inflammation	Granulomatous inflammation	YES	None
8	N	Benign cells	Benign	AIS	No	None
9	Y	Benign cells	Benign	AIS	No	None
10	N	Malignant cells	Adenocarcinoma	Minimal invasive adenocarcinoma	YES	None

ROSE, Rapid On-Site Evaluation; M, male; F, female; Y, yes; N, no; AIS, adenocarcinoma *in situ*; AAH, atypical adenomatous hyperplasia; GGO, ground glass opacity; mm, millimeter; RLL, Right Lower Lobe; LLL, Center Lower Lobe; RUL, Right Upper Lobe; LUL, Center Upper Lobe; POE, point of entry.

The definition of success diagnostic yield is to reflect the concordance between the histopathological or cytopathological diagnosis from BTPNA-obtained specimens and the final surgical pathology.

### Case presentation

Here we present two exemplary cases, cases # 1 and #10, from the 7 successful cases in the present study, representing distinct diagnostic outcomes—one benign and one malignant. (**Table 2**), demonstrating, respectively, **s**uccessful diagnosis of granulomatous inflammation (cryptococcal infection) with no complications, and confirmed minimally invasive adenocarcinoma followed by an uneventful recovery post-segmentectomy.

#### Case 1

**Figure 2:**
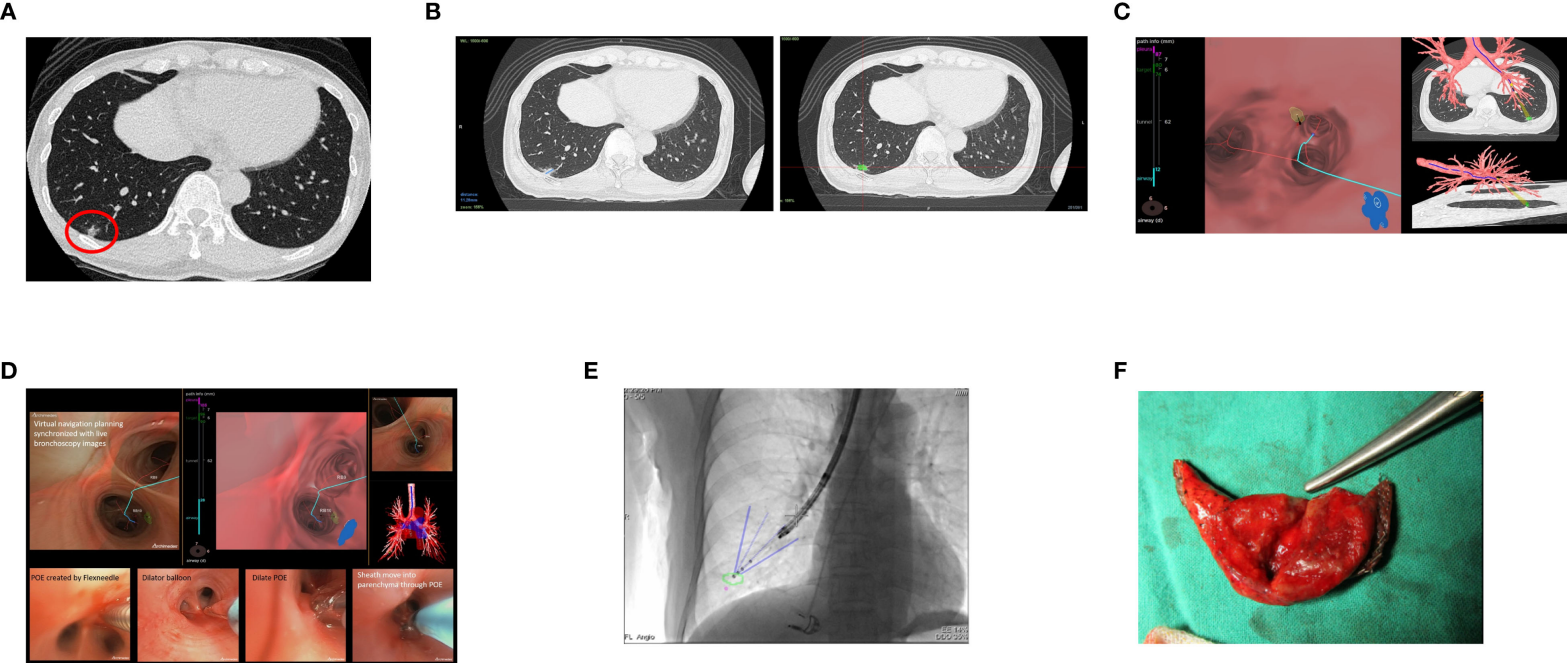
**(A)** Routine low-dose CT screening revealed a 1.1 mm part-solid pulmonary nodule in the right lower lobe (RLL) (red circle). **(B)** The Archimedes™ system localized the nodule in the right lower lobe (RLL), specifically in segment RB10. **(C)** The Archimedes™ system calculated a tunnel length of 61.5 mm and a distance of 7.3 mm from the pleura to the target. **(D)** Images during navigation to reach the target: Virtual navigation planning synchronized with live bronchoscopy images, creation of the point of entry (POE) using the FlexNeedle, dilation of the POE with a balloon dilator, and advancement of the sheath into the parenchyma through the POE. **(E)** CBCT was utilized to confirm precise localization. **(F)** Photo of the resected wedge tissue.

A 53-year-old asymptomatic male civil servant with a history of hypertension, emphysema, and prior pneumothorax (post-surgical intervention in 2006) underwent low-dose CT screening in December 2022, which revealed an 11 mm part-solid pulmonary nodule in the right lower lobe (**Figure 2A**). Repeat CT imaging on April 13, 2023 visualized no significant reduction in nodule size, increasing suspicion of early-stage lung cancer. Using the Archimedes™ navigation system on April 14, 2023, the lesion of 11.3 mm in size was localized in the right bronchopulmonary segment 10 (RB10) (**Figure 2B**). The system calculated a tunnel length of 61.5 mm, and a 7.3 mm distance from pleura to the target (**Figure 2C**, **Table 2**). Throughout BTPNA navigation, fluoroscopy guidance was used to assist in reaching the target, while CT was employed to further confirm the localization (**Figures 2D, E**). A minimally invasive biopsy was then performed which were immediately assessed by a pathologist using ROSE. The preliminary report indicated fibrosis with mild cellular atypia, along with chronic inflammation. Due to still suspicious malignancy in image clinically, VATS was arranged. Prior to surgical resection, 0.2 cc of indocyanine green (ICG) was injected into the lesion via the sheath to assist the surgeon in precise localization. A wedge resection of the RLL was performed (**Figure 2F**). The final postoperative pathology report confirmed no evidence of malignancy, instead revealing chronic granulomatous inflammation with cryptococcal infection.

The procedure was well-tolerated, with stable hemodynamics and no complications. The patient’s postoperative course was uneventful, with a good recovery and complete resolution of symptoms following antifungal treatment.

#### Case 10

**Figure 3:**
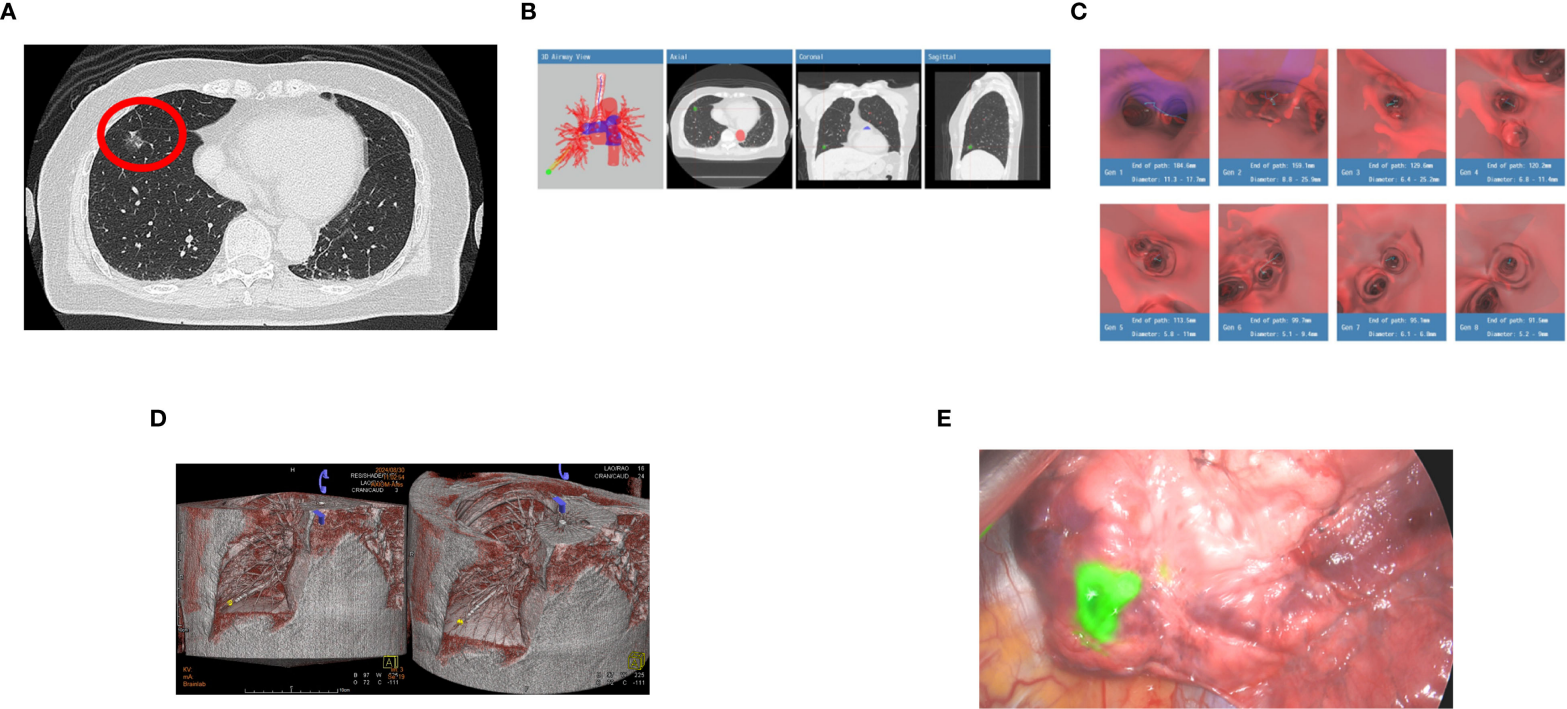
**(A)** A repeated CT confirmed the persistence of the right lower lobe (RLL) ground glass opacity (GGO) without regression (red circle), increasing suspicion of early lung cancer. **(B)** The Archimedes™ system measured the lesion as 15 mm in the right lower lobe (RLL), specifically in segment RB8, with a tunnel length of 82.0 mm and a distance of 17.0 mm from the pleura to the target. **(C)** Images generated by the Archimedes™ system illustrating the calculated path to the target lesion. **(D)** 3D Cone-Beam CT (CBCT) reconstruction during bronchoscopic navigation demonstrates precise guidance of the sheath to the target lesion located in the right lower lobe (RLL). The lesion (highlighted in yellow) is localized accurately, ensuring safe, effective biopsy and subsequent surgical planning. **(E)** 0.2 cc of indocyanine green (ICG) was injected into the lesion via the guide sheath for precise tumor localization of subsequent surgery.

A 73-year-old female (occupation: housewife), non-smoker, and asymptomatic homemaker with no significant past medical history presented for routine evaluation of an incidentally detected 15 mm ground-glass opacity (GGO) in the RLL with intratumoral cystic airspace and mild fissure retraction on CT in May 2023. Additional 4 – 5 mm GGOs in the left lower lobe and right middle lobe were noted, likely focal fibrosis or atypical adenomatous hyperplasia. In July 2023, a repeated CT confirmed the persistence of the RLL GGO without regression, increasing suspicion of early lung cancer (**Figure 3A**). The patient underwent BTPNA on August 30, 2023. The Archimedes™ system located the lesion as 15 mm in RB8, with a tunnel length of 82.0 mm and a distance of 17.0 mm to the pleura (**Figures 3B, C**, **Table 2**). Using BTPNA navigation, a minimally invasive biopsy was performed to obtain tissue samples from the lesion. During navigation, CT imaging was utilized to precisely localize the guidance sheath and lesion (**Figure 3D**). The collected specimen was immediately assessed by ROSE, indicating the presence of malignant cells. Similarly, ICG injection was administered to enhance tumor localization for subsequent surgery (**Figure 3E**). S8 segmentectomy was performed after localizing the lesion by ICG. Blood loss was minimal (50 cc), and the patient remained hemodynamically stable postoperatively. Final pathology confirmed a diagnosis of minimally invasive adenocarcinoma (pT1miN0M0). The patient had an uneventful recovery, and postoperative follow-up revealed good clinical progress without complications.

## Discussion

This study demonstrates the safety and effectiveness of the Archimedes™ navigation system via BTPNA, achieving a 70% diagnostic yield for small peripheral pulmonary lesions (PPLs) under 2 cm without bronchial signs. Among the 10 patients evaluated, no complications were reported, further supporting the procedure’s safety. These results highlight the potential of BTPNA to improve diagnostic accuracy for small, distal PPLs while reducing the need for invasive surgical biopsies and associated risks. Compared with previous vital studies (7, 11, 12), this study especially included the patients with smallest nodule and no bronchial sign for the future integration of BTPNA and radiofrequency ablation (RFA) to provide a seamless solution for non-invasive diagnosis and treatment.

Conventional bronchoscopic biopsy methods have markedly lower diagnostic yields in peripheral lesions lacking an airway path (bronchus sign), often below 30% (13). This limitation arises because standard techniques (radial EBUS or navigation-guided transbronchial biopsy) struggle to reach nodules without a guiding bronchus. BTPNA was specifically developed to overcome this issue by creating a direct transparenchymal access route to the nodule under virtual navigation guidance (10, 13). Emerging high-level evidence demonstrates that BTPNA can substantially improve diagnostic outcomes in these challenging cases. Herth et al. first reported an ~82% diagnostic yield using an Archimedes-guided BTPNA approach in a pioneering trial (7). In our present study, the overall diagnostic yield was 70% with a mean nodule lesion size of 13.9 mm and a median tunnel length range of 55.0 - 61.5 mm.

Besides, the NAVIGATOR study by Hiddinga et al. (12) reported an overall diagnostic yield of 77%, based on a mixed approach that included airway, tunnel, and combined navigation paths. However, for lesions approached solely via the tunnel path specifically, the diagnostic yield was 62% (8 out of 13 cases), with a median nodule size of 25 mm. In comparison, the longer tunnel lengths and smaller mean nodule size in our study suggest an enhanced ability to access deeper, more peripheral, and smaller lesions. In the multicenter study by Sun et al. (11), a biopsy yield of 86.3% was reported across 51 lesions sampled using BTPNA, with a mean tunnel length of 3.3 cm and a mean lesion size of 2.4 cm. While their reported diagnostic yield is higher, it’s worth noting that our study focused on smaller and more technically challenging lesions. Despite these differences, we were still able to achieve a comparable level of diagnostic performance.

In the present study, no complications were observed (0%), highlighting the favorable safety profile and feasibility of the BTPNA procedure. This finding is consistent with several previous studies that have also reported low complication rates for VBN-guided techniques.

For instance, a multicenter study by Sun et al. (11) involving 114 patients who underwent lesion sampling with the Archimedes™ system reported a mean lesion size of 24 mm. Of the 51 lesions sampled using BTPNA, and the remaining with guided TBNA, only two cases of pneumothorax (1.9%) and one case of mild bleeding (1.0%) occurred—none requiring chest tube placement. Although complications were not specifically attributed to either technique, the authors emphasized the safety of BTPNA, even for lesions requiring longer tunnel access.

An earlier study by Harzheim et al. (8) also explored the risks associated with BTPNA. While no significant intraprocedural adverse events occurred, two patients (33.3%) developed postprocedural pneumothorax, one of whom required chest tube drainage. However, these cases involved nodules located very close to the pleura (1 mm and 9 mm, respectively), highlighting the importance of preprocedural planning and patient selection, especially when dealing with subpleural lesions.

Additionally, Verga et al. (14) evaluated 96 patients with advanced lung disease undergoing VBN-guided sampling. The mean lesion size was 23 mm, with BTPNA performed in only three cases. Most patients underwent conventional VBN-guided transbronchial approaches in combination with radial EBUS and fluoroscopy. The overall complication rate was 7%, including 1% pneumothorax, 3% significant bleeding, and 3% respiratory failure.

In contrast, traditional CT-guided transthoracic needle aspiration is associated with higher risks, including pneumothorax rates of approximately 15 – 25% and chest tube placement in 4 – 6% of cases (15). BTPNA may offer a less invasive alternative with fewer complications.

BTPNA may serve as a critical platform for advancing novel bronchoscopic therapies aimed at treating peripheral lung lesions (PPLs), including RFA, microwave ablation, bronchoscopic thermal vapor ablation (BTVA), and cryotherapy. Changhao Zhong and colleagues reported promising one-year results for transbronchial RFA, with a complete ablation rate of 90.48% and an intrapulmonary progression-free survival rate of 88.89% (16). Additionally, Daniel P. Steinfort and his team demonstrated the efficacy of BTVA in six patients, observing up to 99% tumor necrosis within the ablation zones (17). Looking forward, BTPNA—particularly when integrated with other ablation strategies—holds strong potential as a central platform in the treatment of PPLs.

## Limitations

This study has several limitations. The small sample size and single-center design restrict the generalizability of the findings to broader patient populations. Additionally, potential selection bias—such as the exclusion of high-risk patients with severe COPD, pulmonary fibrosis, or coagulopathy—may limit the applicability of the results to real-world clinical scenarios involving peripheral lung lesions (PPLs).

## Conclusions

The Archimedes™ navigation system-guided BTPNA has demonstrated promise as a minimally invasive approach for diagnosing small and difficult-to-access peripheral pulmonary lesions (PPLs) without bronchus signs, achieving a 70% diagnostic yield without any reported complications in the current study. However, larger prospective multicenter trials are needed to confirm these findings, further establish the system’s efficacy, enhance safety protocols, and expand its clinical applicability.

## Data Availability

The original contributions presented in the study are included in the article/supplementary material. Further inquiries can be directed to the corresponding authors.
